# Release of Iron-Loaded Ferritin in Sodium Iodate-Induced Model of Age Related Macular Degeneration: An In-Vitro and In-Vivo Study

**DOI:** 10.3390/antiox10081253

**Published:** 2021-08-05

**Authors:** Ajay Ashok, Suman Chaudhary, Aaron S. Wise, Neil A. Rana, Dallas McDonald, Alexander E. Kritikos, Ewald Lindner, Neena Singh

**Affiliations:** 1 Department of Pathology, School of Medicine, Case Western Reserve University, Cleveland, OH 44106, USA; axa864@case.edu (A.A.); sxc1351@case.edu (S.C.); asw80@case.edu (A.S.W.); nar66@case.edu (N.A.R.); djm259@case.edu (D.M.); aek103@case.edu (A.E.K.); 2 Department of Ophthalmology, Medical University of Graz, Auenbruggerplatz 4, 8036 Graz, Austria; ewald.lindner@medunigraz.at

**Keywords:** ferritin, exosomes, age-related macular degeneration, lysosomes, sodium iodate

## Abstract

To evaluate the role of iron in sodium iodate (NaIO_3_)-induced model of age-related macular degeneration (AMD) in ARPE-19 cells in-vitro and in mouse models in-vivo. ARPE-19 cells, a human retinal pigment epithelial cell line, was exposed to 10 mM NaIO_3_ for 24 h, and the expression and localization of major iron modulating proteins was evaluated by Western blotting (WB) and immunostaining. Synthesis and maturation of cathepsin-D (cat-D), a lysosomal enzyme, was evaluated by quantitative reverse-transcriptase polymerase chain reaction (RT-qPCR) and WB, respectively. For in-vivo studies, C57BL/6 mice were injected with 40 mg/kg mouse body weight of NaIO_3_ intraperitoneally, and their retina was evaluated after 3 weeks as above. NaIO_3_ induced a 10-fold increase in ferritin in ARPE-19 cells, which co-localized with LC3II, an autophagosomal marker, and LAMP-1, a lysosomal marker. A similar increase in ferritin was noted in retinal lysates and retinal sections of NaIO_3_-injected mice by WB and immunostaining. Impaired synthesis and maturation of cat-D was also noted. Accumulated ferritin was loaded with iron, and released from retinal pigmented epithelial (RPE) cells in Perls’ and LAMP-1 positive vesicles. NaIO_3_ impairs lysosomal degradation of ferritin by decreasing the transcription and maturation of cat-D in RPE cells. Iron-loaded ferritin accumulates in lysosomes and is released in lysosomal membrane-enclosed vesicles to the extracellular milieu. Accumulation of ferritin in RPE cells and fusion of ferritin-containing vesicles with adjacent photoreceptor cells is likely to create an iron overload, compromising their viability. Moreover, reduced activity of cat-D is likely to promote accumulation of other cellular debris in lysosomal vesicles, contributing to AMD-like pathology.

## 1. Introduction

Retinal iron accumulation and iron-mediated oxidative stress are considered risk factors for age-related macular degeneration (AMD), a leading cause of blindness in developed countries [1,2]. Several etiological factors contribute to the development of AMD, including genetic, metabolic, and environmental. The multifactorial nature of this disorder has been a significant challenge in developing effective therapeutic options. Dysfunction of the retinal pigment epithelial (RPE) cell layer is a consistent observation and is clinically identified with the accumulation of extracellular amorphous material, known as drusen, between the RPE cell layer and inner layer of the Bruch’s membrane. Accumulation of phagocytosed material including break-down products of photoreceptor outer segments (POS) results in the loss of metabolic support and eventual death of RPE and photoreceptor cells, resulting in irreversible vision loss [3,4,5,6,7,8].

RPE cells are a monolayer of specialized epithelial cells that form the outer blood–retinal barrier (BRB) on their basolateral side and interact closely with the photoreceptor cell layer on the apical side [9,10]. The BRB regulates the exchange of nutrients and metabolites between peripheral blood and the retina, and interaction with photoreceptors facilitates the essential function of phagocytosis and turnover of POS. Each day, ~10% of the total volume of photoreceptor cells is phagocytosed, and roughly the same amount is regenerated. Since RPE cells are post-mitotic, disposal of phagocytosed material is necessary to avoid waste build-up, including an efficient autophagosomal–lysosomal machinery. In fact, RPE is the most active phagocytic cell in the body, and perturbation of this function results in retinal disease, including AMD [6,11].

Features typical of AMD have been reproduced by the accumulation of iron in the human retina and in experimental models [1,12]. In hemochromatosis and aceruloplasminemia, hereditary human disorders associated with systemic iron overload, iron collects in the retina despite an intact BRB, resulting in AMD-like pathology [1,13]. Likewise, administration of intravenous iron for refractory anemias leads to retinal iron accumulation, and is a risk factor for AMD [14]. The retina of aged individuals has significantly more iron than young adults [15], supporting a significant role of iron in AMD pathogenesis. In experimental mouse models, absence of ceruloplasmin and hephaestin, ferroxidases necessary for iron export, results in retinal iron overload and AMD-like pathology [2,16], reinforcing the involvement of iron. In cultured ARPE-19 cells, exposure to iron decreases their phagocytic activity and lysosomal function [17,18], a characteristic typical of AMD.

Sodium iodate (NaIO_3_), an oxidizing agent, has been used extensively to replicate human AMD in cell and animal models. Toxicity by NaIO_3_ is specific to RPE cells, and low doses cause patchy loss of tight junctions, disrupt outer and inner photoreceptor segments, and cause accumulation of degradation products of POS typical of AMD [19]. The mechanisms underlying these changes have been explored extensively, with each report emphasizing the significance of a specific biochemical pathway [20,21,22,23,24,25]. Interestingly, partial reversal of NaIO_3_-induced AMD in animal models by the iron chelator deferiprone [26] suggests a significant role of iron in AMD pathogenesis. In this report, we evaluated the role of iron in NaIO_3_-induced AMD in C57BL/6 mice and human ARPE-19 cells.

## 2. Methods

### 2.1. Ethics Statement

All animal experiments were approved by the Institutional Animal Care and Use Committee at the School of Medicine, Case Western Reserve University (ethical protocol number: 2015–0027), and were conducted in accordance with the guidelines of the Association for Research in Vision and Ophthalmology on the use of animals in research. Animals were housed in the Association of Assessment and Accreditation of Laboratory Animal Care International (AAALAC)-approved Animal Resource Center (ARC) at Case Western Reserve University School of Medicine. All methods adhered to the ARVO Statement for the Use of Animals in Ophthalmic and Vision Research.

### 2.2. Antibodies and Chemicals

The list of antibodies used in the study are provided in Supplementary Table S1. Sodium iodate (NaIO_3_) and all other chemicals were obtained from Sigma Aldrich, Milwaukee, WI, USA (S4007-100G). Cathepsin-D siRNA was obtained from Santa Cruz, Dallas, TX, USA (sc-29239). Lipofectamine RNAiMax was from Invitrogen, (Fulfilled by Supply Center, Cleveland, OH, USA).

### 2.3. Animals

C57BL/6J mice (5 per group) were injected intraperitoneally with NaIO_3_ (40 mg/kg mouse body weight) or equal volume of saline, and sacrificed after 3 weeks. Retinal lysates were processed for Western blotting (WB), and thin sections from fixed tissue were evaluated by immunohistochemistry. Retinal tissue and a piece of brain tissue from the frontal cortex of 5 mice from each experimental group were pooled, and an equal amount of protein was evaluated for ferritin expression by WB.

### 2.4. Cell Culture

Human retinal pigmented epithelial cell line (ARPE-19; ATCC^®^ CRL-2302™) was used from passage 4 to 20. The phenotype of these cells was confirmed before use by probing cell lysates for RPE 65 by WB, a marker for RPE cells [27]. Cultures were maintained in Dulbecco’s Modified Eagle Medium/Nutrient Mixture F-12 (DMEM/F12) supplemented with 10% fetal bovine serum (FBS), 100 U/mL penicillin, and 100 µg/mL streptomycin. The medium was changed every 2 days, and sub-confluent cultures in 1% FBS were treated with 10 mM NaIO_3_ for 24 h before processing for WB and immunostaining. To silence cat-D, the cells were transfected with cat-D-specific or the corresponding scrambled siRNA using Lipofectamine RNAiMax as per manufacturer’s instructions. Desired downregulation of cat-D was confirmed by WB.

### 2.5. Sodium Dodecyl Sulfate Polyacrylamide Gel Electrophoresis (SDS-PAGE) and Western Blotting

Protein lysates were fractionated on SDS-PAGE and analyzed by WB as described earlier [28,29]. Proteins of interest were detected in transferred lysates with specific antibodies followed by species-specific horseradish peroxidase (HRP)-conjugated secondary antibody (Supplementary Table S1). Specific bands were visualized on an X-ray film with enhanced chemiluminescence. Full blots and their details are provided in Supplementary Figure S1.

### 2.6. Quantitative Reverse-Transcriptase Polymerase Chain Reaction (RT-qPCR)

Total RNA was isolated from cells using RNAqueous^®^-Micro Kit (AM1931, Invitrogen (Fulfilled by Supply Center, Cleveland, OH, USA) according to manufacturer’s instructions, and PCR reactions were primed with BrightGreen 2X QPCR MasterMix (MasterMix-R) (Applied Biological Materials Inc, Ferndale, WA, USA). The mRNA of interest was amplified using the following primers: Hu cat-D-F: CTGCACAAGTTCACGTCCAT-3′, Hu cat-D-R: TTCTGCTGCATCAGGTTGTC-3′ (Accession No.: NM_001909.5). Hu GAPDH-F: GAGTCAACGGATTTGGTCGT-3′, Hu GAPDH-R: GGTGCCATGGAATTTGCCAT-3′ (Accession No.: NM_001289745.3). All primers were obtained from Integrated DNA Technologies, Coralville, IA, USA.

### 2.7. Immunostaining

Immunostaining of fixed cells and retinal tissue sections was performed essentially as described [30,31]. Following treatments, ARPE-19 cells were washed with 1 × PBS, fixed using 4% paraformaldehyde, blocked using 2% bovine serum albumin (BSA) and co-immunostained. The cells were incubated with antibodies against LC3II (produced in rabbit) and ferritin (produced in goat) and were detected using anti-rabbit secondary antibody conjugated with Alexa Fluor 546 (red) and anti-goat secondary antibody conjugated with Alexa Fluor 488 (green), respectively. Co-immunostained ARPE-19 cells were incubated with antibodies against LAMP-1 (produced in rabbit) and ferritin (produced in goat) and were detected using anti-rabbit secondary antibody conjugated with Alexa Fluor 546 (red) and anti-goat secondary antibody conjugated with Alexa Fluor 488 (green), respectively. Controls were treated with no primary antibodies but reacted with both secondary antibodies (Supplementary Figure S2). Staining of retinal tissue sections was performed similarly, and serial sections were used as controls for staining with secondary antibody only (Supplementary Figure S3).

Hoechst was used to stain the nuclei in all immunostaining experiments. Stained samples were imaged with Leica inverted microscope (DMi8). Each experiment was repeated three times, and a representative image from 10 different fields is shown.

### 2.8. Perls’ Staining

Perls’ staining was performed as described earlier [32]. Cells and tissue sections were rinsed, let to react with a mixture of ferrocyanide–HCl for 10 min, and counter-stained with filtered neutral red stain for 1 min. Stained samples were rapidly dehydrated in absolute alcohol and mounted for imaging with an upright microscope (Leica Microscope Imaging System; Leica, Wetzlar, Germany). Each experiment was repeated 3 times, and a representative image from 10 different fields is shown.

### 2.9. Statistical Analysis

Densitometry of images was performed with UN-SCAN-IT gels (version 6.1) software (Silk Scientific, Orem, UT, USA) and Image J software (Version: 1.53K, National Institutes of Health, Bethesda, MD, USA). All data were statistically analyzed by GraphPad Prism software (Version 5.0, GraphPad Software Inc., San Diego, CA, USA) and are shown as mean ± SEM. Significant differences between control and experimental samples were determined by the Student’s unpaired *t*-test or Two-way ANOVA. Differences were considered statistically significant at *p* ≤ 0.05.

## 3. Results

### 3.1. Ferritin Is Upregulated by Sodium Iodate in ARPE-19 Cells

**Figure 1:**
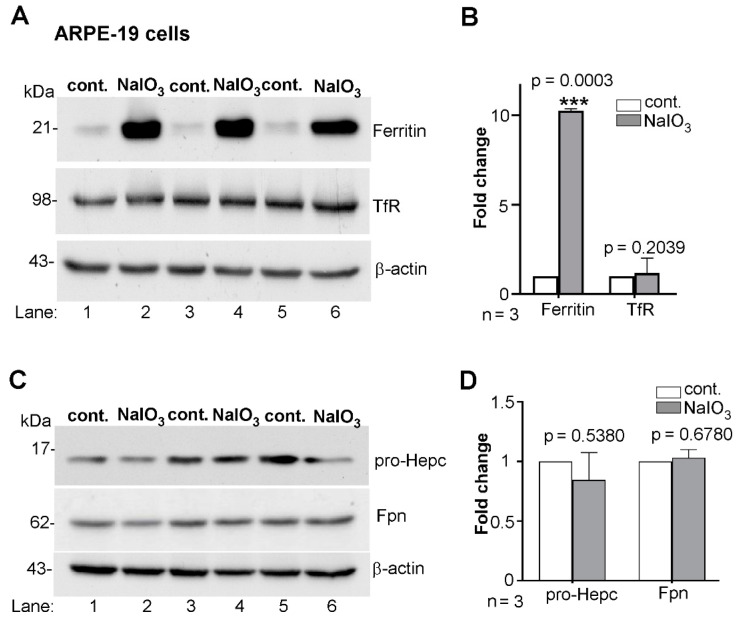
NaIO_3_ upregulates ferritin in ARPE-19 cells without altering other iron modulating proteins. (**A**) Probing of lysates from ARPE-19 cells exposed to 10 mM NaIO_3_ for 24 h for ferritin revealed significant upregulation relative to vehicle treated controls (lanes 2, 4, 6 vs. 1, 3, 5). Re-probing of the same membrane for transferrin receptor (TfR) showed no significant change in their levels between the experimental and control groups (lanes 2, 4, 6 vs. 1, 3, 5). (**B**) Densitometry after normalization with β-actin showed a 10-fold increase in ferritin and minimal change in TfR by NaIO_3_ treatment relative to controls. (**C**) Probing of lysates from a similar experimental setup for pro-hepcidin and Fpn showed no significant change in their levels by NaIO_3_-treatment relative to controls (lanes 2, 4, 6 vs. 1, 3, 5). (**D**) Densitometry after normalization with β-actin shows non-significant changes in the levels of pro-hepcidin and Fpn between experimental and control samples. Full blots and their details are provided in Supplementary Figure S1. Values are means ± SEM of the indicated *n*. *** *p* ≤ 0.001.

To assess the role of iron in NaIO_3_-mediated AMD-like changes in ARPE-19 cells, three independent sets of sub-confluent cultures were exposed to 10 mM NaIO_3_ for 24 h, and the expression of major iron modulating proteins ferritin, transferrin receptor (TfR), hepcidin, and ferroportin (Fpn) was evaluated by WB. Probing for ferritin revealed significantly increased levels in NaIO_3_-treated relative to vehicle-treated controls in all three sets (Figure 1A, lanes 2, 4, and 6 vs. 1, 3, and 5; Figure 1B). Surprisingly, re-probing for transferrin receptor (TfR) showed no change in its levels despite the increased levels of ferritin (Figure 1A, lanes 2, 4, and 6 vs. 1, 3, and 5; Figure 1B). Normally, an increase in intracellular iron is expected to downregulate the TfR, an iron uptake protein, to block additional uptake of iron.

Another consequence of intracellular accumulation of iron is decreased expression of hepcidin, the master regulator of iron homeostasis, thereby sparing Fpn and increasing iron export. ARPE-19 cells are known to synthesize hepcidin [33] and regulate their iron content and its transport from the peripheral circulation to the retina by modulating the expression of Fpn. Probing of NaIO_3_-treated ARPE-19 cell lysates for pro-hepcidin, a precursor of hepcidin most prominent in cell lysates, showed no change in its levels relative to vehicle treated controls (Figure 1C, lanes 2, 4, and 6 vs. 1, 3, and 5; Figure 1D). Likewise, there was little change in Fpn (Figure 1C, lanes 2, 4, and 6 vs. 1, 3, and 5; Figure 1D). These observations suggest either of two possibilities: upregulation of ferritin is unrelated to iron accumulation, or the accumulated iron is sequestered in ferritin and unable to alter the intracellular labile iron pool (LIP).

### 3.2. Ferritin Co-Localizes with the Autophagosomal Marker LC3II

Ferritin stores iron in the relatively non-toxic, ferric form to protect cells from the relatively toxic ferrous iron and requires degradation for the release of iron when necessary. This is mediated by nuclear receptor coactivator 4 (NCOA4), an autophagy receptor which delivers iron-loaded ferritin to autophagosomes. These fuse with lysosomes, where ferritin is degraded mainly by cathepsin-D (cat-D), a lysosomal enzyme [34]. The iron released from ferritin is transported to the cytosol to replenish the LIP for metabolic purposes. Since the LIP of NaIO_3_-treated cells does not appear to change, it is possible that either the cells lack NCOA4 and are unable to deliver ferritin to autophagosomes, or the degradation of ferritin in lysosomes is impaired, resulting in its accumulation.

**Figure 2:**
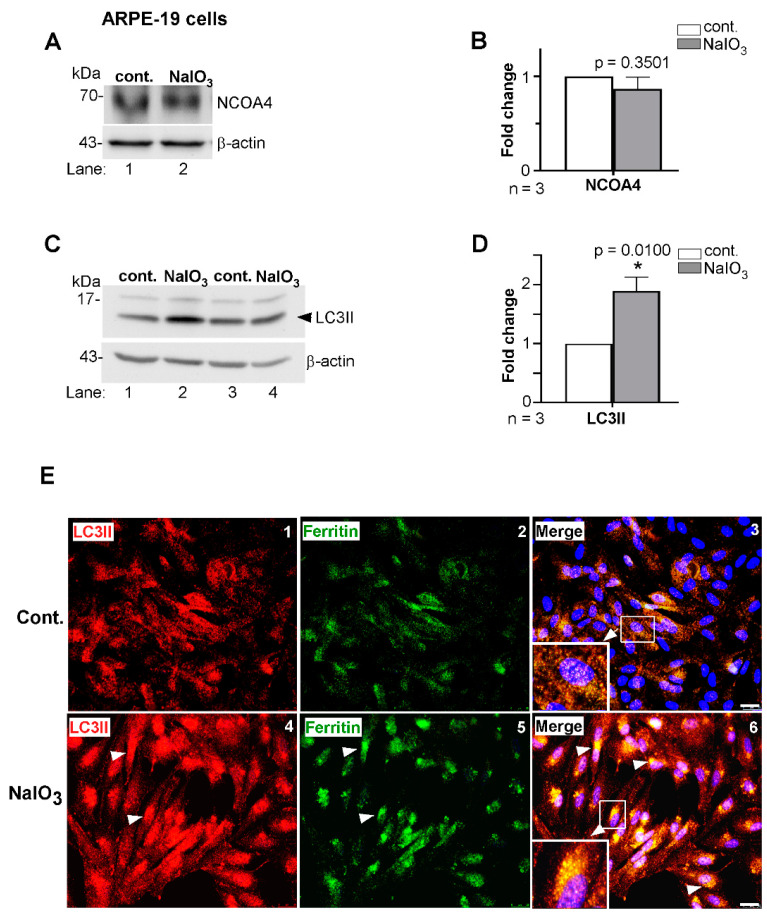
Ferritin accumulates in autophagosomes in NaIO_3_-treated cells. (**A**) Probing of lysates from NaIO_3_ treated and control cells as above for nuclear receptor coactivator 4 (NCOA4) showed no difference in expression between the experimental and control groups (lane 2 vs. 1). (**B**) Densitometry after normalization with β-actin showed no significant change in the levels of NCOA4 in NaIO_3_-treated cells relative to controls. (**C**) Probing of lysates from a similar experimental set-up for LC3II showed increased expression in NaIO_3_-treated cells relative to controls (lanes 2, 4 vs. 1, 3). (**D**) Densitometry after normalization with β-actin showed a 2-fold increase in LC3II in NaIO_3_-treated cells relative to controls. Full blots and their details are provided in Supplementary Figure S1. Values are means ± SEM of the indicated *n*. * *p* ≤ 0.05. (**E**) Co-immunostaining of ARPE-19 cells with goat anti-ferritin and rabbit anti-LC3II followed by species-specific Alexa Fluor 488 (**green**)- and Alexa Fluor 546 (**red**)-conjugated secondary antibodies showed increased co-localization (**white arrows**) of the two proteins in NaIO_3_-treated cells relative to untreated controls (panel 6 vs. 3). Cells treated with only secondary antibodies showed no reaction (Supplementary Figure S2). Scale bar: 25 µm.

To evaluate this possibility, we assessed levels of NCOA4 and LC3II, the latter a marker of autophagosomes. Thus, lysates prepared from ARPE-19 cells treated with NaIO_3_ or vehicle for 24 h were processed for WB and probed for NCOA4. No difference was noted in the levels of NCOA4 in two independent samples of NaIO_3_-treated cells relative to controls (Figure 2A, lanes 2 vs. 1; Figure 2B). A similar evaluation of LC3II, an autophagosomal marker, showed a significant increase in expression in NaIO_3_-treated cells relative to controls (Figure 2C, lanes 2 and 4 vs. 1 and 3; Figure 2D), suggesting efficient formation and maturation of autophagosomes [35]. Co-immunostaining of fixed, permeabilized cells for ferritin and LC3II showed co-localization in perinuclear autophagosomes, which were more prominent in NaIO_3_-treated cells relative to controls (Figure 2E, panel 6 vs. 3).

Together, these results suggest that ferritin is delivered efficiently to LC3II-positive autophagosomes for subsequent fusion and degradation in lysosomes.

### 3.3. Ferritin Accumulates in Lysosomes and Is Released in LAMP-1 Positive Vesicles

**Figure 3:**
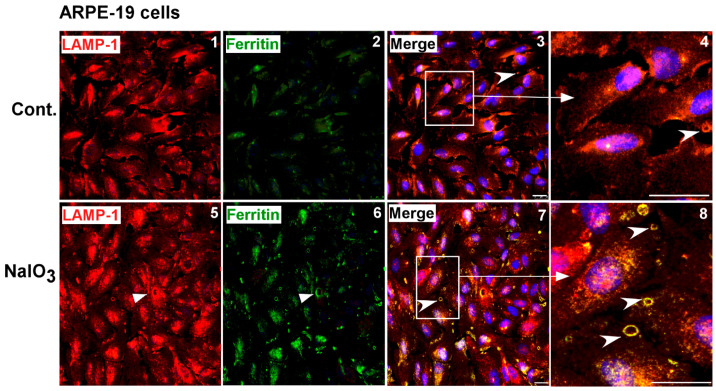
NaIO_3_ induces release of ferritin in lysosomal membrane-enclosed vesicles. Co-immunostaining of ARPE-19 cells with goat anti-ferritin and rabbit anti-LAMP-1 antibodies followed by species-specific secondary antibodies conjugated with Alexa Fluor 546 (**red**) and Alexa Fluor 488 (**green**) showed co-localization in intracellular (panels 7, 8, arrowhead) and extracellular (panels 7, 8 vs. panels 3, 4, arrowhead) vesicles in NaIO_3_-treated cells relative to controls. Extracellular vesicles positive for LAMP-1 were also seen in vehicle-treated controls (panels 3, 4, arrowhead). Cells treated with only secondary antibodies showed no reaction (Supplementary Figure S2). Experiments shown in Figure 2E and Figure 3 were conducted simultaneously to maintain scientific rigor and had the same controls. Scale bar: 25 µm.

To evaluate whether autophagosomes fuse with lysosomes and deliver iron-loaded ferritin for degradation, NaIO_3_- and vehicle-treated cells were fixed and co-immunostained for ferritin and the lysosomal membrane protein LAMP-1. Surprisingly, several vesicular structures positive for ferritin and LAMP-1 were detected in the intercellular space (Figure 3, panels 7, 8). In some cells, ferritin was detected intracellularly in LAMP-1 positive lysosomes (Figure 3, panels 7, 8, arrow). Similar structures, though significantly less in number and lacking in ferritin, were detected in vehicle-treated controls (Figure 3, panels 3, 4).

**Figure 4:**
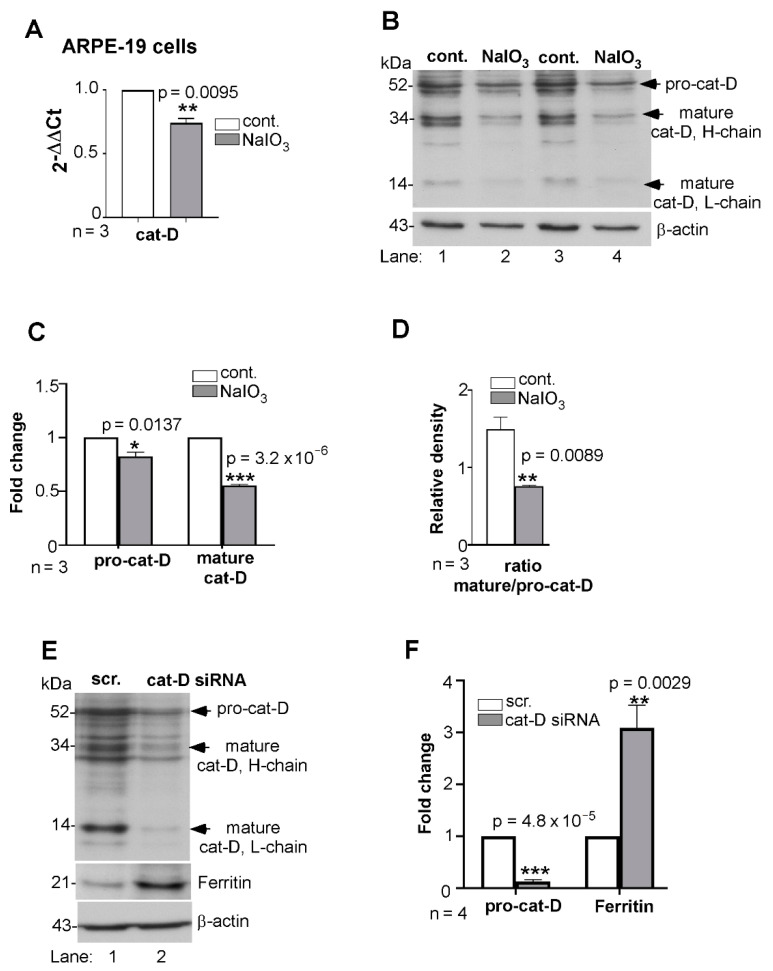
Synthesis and maturation of cathepsin-D is compromised by NaIO_3._ (**A**) Quantitative RT-qPCR for cat-D showed 0.75-fold downregulation in lysates from NaIO_3_-treated ARPE-19 cells relative to controls. Human GAPDH was amplified in parallel. (**B**) Probing of NaIO_3_-treated cell lysates for cat-D showed decreased expression of pro-cat-D and its mature form relative to controls (lanes 2, 4 vs. 1, 3). (**C**) Densitometry after normalization with β-actin showed 0.7- and 0.5-fold decreases in the levels of pro-cat-D and mature cat-D in NaIO_3_-treated samples relative to the controls. (**D**) The densitometry ratio of mature cat-D relative to pro-cat-D was significantly lower in NaIO_3_-treated cell lysates relative to control. (**E**) Probing of ARPE-19 cell lysates for cat-D showed the expected reactivity of the pro-cat-D form at 52 kDa in control cells transfected with scrambled siRNA, and minimal reaction in cells exposed to cat-D siRNA (lane 2 vs. 1). This further led to lowered levels of mature cat-D levels in cat-D siRNA-treated cell lysates. Probing for ferritin showed significant upregulation in the absence of cat-D relative to control (lanes 2 vs. 1). (**F**) Quantification by densitometry after normalization with β-actin showed 2.7-fold upregulation of ferritin due to downregulation of cat-D. Full blots and their details are provided in Supplementary Figure S1. Values are means ± SEM of the indicated *n*. * *p* ≤ 0.05; ** *p* ≤ 0.01; *** *p* ≤ 0.001.

The release of ferritin in lysosomal membrane-enclosed vesicles suggested impaired degradation and exocytosis of accumulated ferritin. To evaluate whether cat-D, an aspartic endo-protease responsible for the turnover of a variety of substrates including ferritin, is responsible [36,37], synthesis of cat-D was evaluated by quantitative reverse-transcriptase polymerase chain reaction (RT-qPCR). Total mRNA was isolated from triplicate cultures of NaIO_3_- and vehicle-treated cells and amplified using cat-D-specific primers. A significant transcriptional downregulation of cat-D was observed in NaIO_3_-treated cells relative to controls (Figure 4A). Subsequent evaluation of two independent samples by WB revealed significantly reduced levels of pro-cat-D, and additionally, decreased maturation of pro-cat-D to its mature form (Figure 4B, lanes 2 and 4 vs. 1 and 3; Figure 4C). This finding was further validated by estimating the ratio of mature to pro-cat-D using the immunoblotting results. The ratio of the intensities clearly indicated a diminished maturation of cat-D from its pro form in NaIO_3_-treated cells relative to controls (Figure 4D). To further confirm that cat-D downregulation inhibited ferritin turnover, ARPE-19 cells were transfected with cat-D-specific siRNA and the lysates were let to immunoreact with ferritin. Successful downregulation of cat-D resulted in increased accumulation of ferritin relative to scrambled siRNA-treated controls (Figure 4E, lanes 2 vs. 1; Figure 4F), suggesting ferritin to be one of the substrates processed by cat-D in these cells.

### 3.4. Ferritin Accumulates in the Retina of Sodium-Iodate-Injected Mice

**Figure 5:**
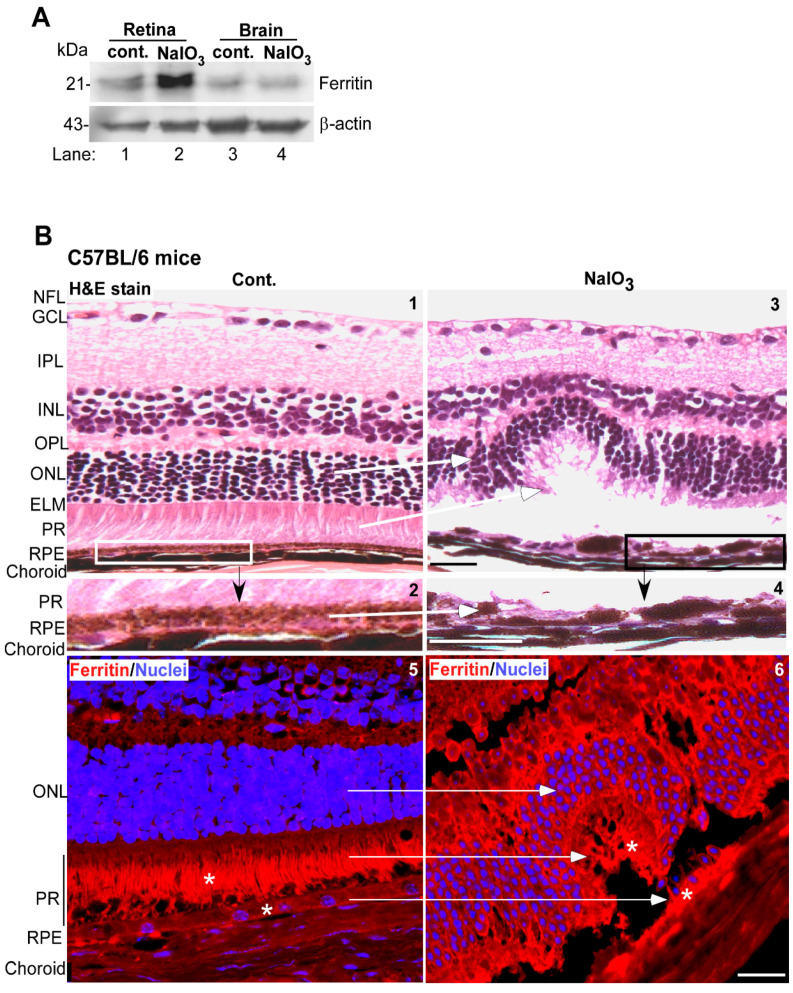
NaIO_3_ upregulates ferritin and disrupts retinal cell layers in mouse models. (**A**) Probing of retinal and brain lysates from NaIO_3_ and control mice for ferritin showed significantly higher levels in retinal samples (lane 2 vs. 1) relative to controls. Brain samples from the same mice showed a similar level of ferritin (lane 4 vs. 3). (Tissue from 5 mice was pooled for this experiment). The membranes were re-probed for β-actin as a loading control. Full blots and their details are provided in Supplementary Figure S1. (**B**) Hematoxylin and Eosin (H&E) staining of mouse retina showed well-defined layers in control mice (panel 1). The boxed area is enlarged in panel 2. Mice administered NaIO_3_ showed disruption of the RPE and photoreceptor cell layers (panel 3). Scale bar: 25 µm. The boxed area is enlarged in panel 4. Scale bar: 25 µm. Immunoreaction for ferritin showed increased expression in the RPE and photoreceptor cell layers of NaIO_3_-treated mice relative to controls (panel 6 vs. 5, marked by *). Scale bar: 25 µm. A serial section treated with rabbit IgG and Alexa Fluor 546-conjugated secondary antibody (red) showed no reaction (Supplementary Figure S3). RPE: retinal pigment epithelium; PR: photoreceptors; ELM: external limiting membrane; ONL: outer nuclear layer; OPL: outer plexiform layer, INL: inner nuclear layer, IPL: inner plexiform layer, GCL: ganglion cell layer; NFL: nerve fiber layer.

The in-vitro findings in ARPE-19 cells were further confirmed in mouse models. Wild-type C57BL/6 mice were given a single intraperitoneal injection of NaIO_3_ (40 mg/kg mouse body weight), and evaluated after 3 weeks. Controls received equal volume of saline. Both the retinal tissue and a piece of brain tissue from the frontal cortex of five mice were pooled, and an equal amount of protein was evaluated for ferritin levels by WB. As in ARPE-19 cells, a significant increase in ferritin was observed in retinal samples from NaIO_3_-treated mice relative to controls (Figure 5A, lane 2 vs. 1). No change was observed in the brain tissue of the same mice (Figure 5A, lane 4 vs. 3), confirming previous reports that NaIO_3_ affects the retina specifically.

Hematoxylin and Eosin (H&E) staining of fixed retinal sections from NaIO_3_ and vehicle-treated controls showed well-defined retinal layers in vehicle-treated controls (Figure 5B, panel 1). The RPE and photoreceptor cell layer were uniform and well-defined as expected (Figure 5B, panel 2). In retinal sections from NaIO_3_-treated mice, the RPE cell layer was distorted, and so was the photoreceptor cell layer (Figure 5B, panels 3 and 4, white arrows) [22]. Immunostaining for ferritin showed a reaction in RPE cells and a prominent signal in photoreceptor outer segments (Figure 5B, panel 5, marked with *). In NaIO_3_-treated sections, a prominent reaction for ferritin was noted in the RPE cell layer and the photoreceptors (Figure 5B, panel 6, marked with *).

### 3.5. Accumulated Ferritin Is Loaded with Iron

**Figure 6:**
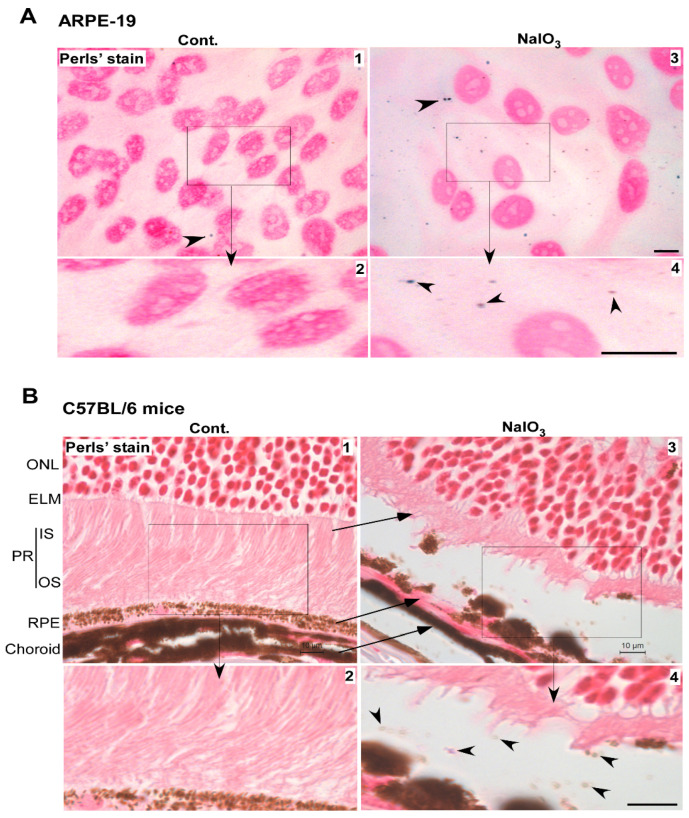
Iron-loaded vesicles are released in the extracellular milieu by NaIO_3_ treatment. (**A**) Staining with Perls’ reagent showed several blue dots intracellularly and extracellularly in NaIO_3_-treated ARPE-19 cells (panel 3, arrowhead). Scale bar: 10 µm. Boxed area is enlarged in panel 4. Scale bar: 10 µm. A rare blue dot was also noted in control cells (panel 1, arrowhead). Boxed area is enlarged in panel 2. Scale bar: 10 µm. (**B**) Perls’ reaction of retinal sections from NaIO_3_-treated mice showed a positive reaction in the space between RPE and PR cells (panel 3, arrowhead), better visualized in the enlarged boxed area (panel 4, arrowheads). Scale bar for panel 3: 10 µm. Scale bar for panel 4: 10 µm. RPE: retinal pigment epithelium; PR: photoreceptors; OS: outer segment of photoreceptors; IS: inner segment of photoreceptors; ELM: external limiting membrane; ONL: outer nuclear layer.

To evaluate whether the accumulated ferritin is loaded with iron, ARPE-19 cells treated with NaIO_3_ or vehicle were let to react with the Perls’ reagent, and the nuclei counterstained with neutral red. Several blue dots indicating positive reaction with ferric iron were detected in the extracellular space and intracellularly in NaIO_3_-treated cells (Figure 6A, panels 3 and 4). A rare blue dot was also detected in control cells treated with vehicle (Figure 6A, panels 1 and 2). A similar evaluation of retinal sections from NaIO_3_-treated mice showed Perls’ positive vesicles in the space between the RPE and photoreceptor cell layer (Figure 6B, panels 3 and 4). No reaction was detected in vehicle-treated samples (Figure 6B, panels 1 and 2).

Together, these observations suggest that NaIO_3_-induced toxicity is mediated by the accumulation of iron-loaded ferritin in lysosomes and its release in the surrounding tissue in membrane-enclosed vesicles.

## 4. Conclusions

**Figure 7:**
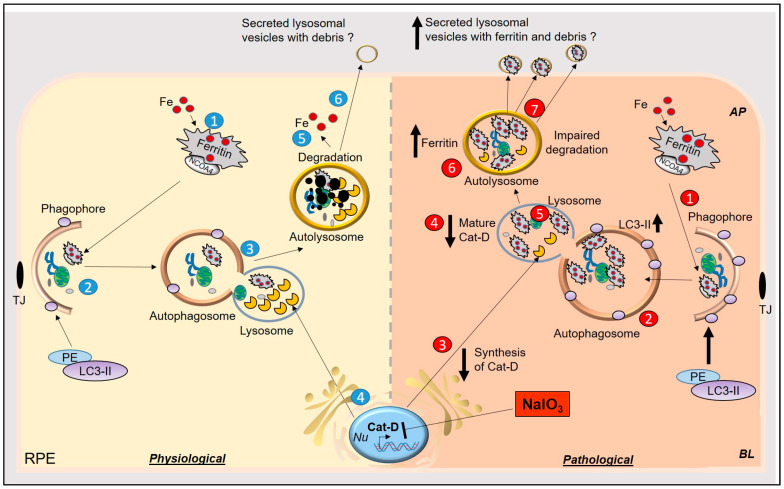
Graphical representation of NaIO_3_-induced model of AMD in-vitro and in-vivo. *Physiological*: (1) Iron-loaded ferritin is transported to the phagophore by NCOA4, (2) for degradation with the rest of the cell debris. Levels of LC3II increase with the maturation of phagophore to autophagosome. (3) Autophagosome fuses with lysosomes, (4) where lysosomal enzymes, including cat-D, degrade ferritin and cellular debris. (5) Iron from ferritin is released in the cytosol to be utilized for metabolic purposes. (6) Other debris undergoes autolysis or is secreted via an unconventional lysosomal secretory pathway to the extracellular milieu. *Pathological:* (1) In NaIO_3_-induced AMD, iron-loaded ferritin is delivered to the phagophore, (2) which matures into autophagosomes. Levels of LC3II are increased because of accumulation of debris in autophagosomes. (3 and 4) Synthesis and maturation of cat-D are impaired by NaIO_3_, (5) resulting in the accumulation of iron-loaded ferritin in lysosomes and (6) autolysosomes. (7) Iron-loaded ferritin is secreted in lysosomal vesicles in the extracellular milieu, which is probably internalized by the adjacent photoreceptors, resulting in iron-mediated oxidative stress.

We report dysfunction of lysosomes as a significant contributing factor in AMD-like pathology mediated by NaIO_3_ in cell and mouse models. Decreased synthesis and maturation of cat-D induced by NaIO_3_ resulted in the accumulation of iron-loaded ferritin in lysosomes, which was released from cells in lysosomal membrane-derived vesicles. In mouse models, RPE and photoreceptor cell layers were disorganized, and the retina showed accumulation of ferritin as in cell models. These results implicate iron in NaIO_3_-induced experimental models of AMD, and possibly in human AMD as well because NaIO_3_ reproduces several features of this disorder (Figure 7).

Our observations support previous reports implicating iron in AMD pathogenesis, but suggest a distinct mechanism of retinal iron overload [2,16]. The almost 10-fold increase in iron-loaded ferritin in RPE cells in-vitro and in-vivo is due to dysfunctional lysosomes that are unable to degrade iron-loaded ferritin to release the bound iron [38]. This is supported by no change in the cellular labile iron pool as indicated by minimal change in the expression of major iron modulating proteins such as hepcidin, Fpn, and TfR. Normally, hepcidin is downregulated by increase in intracellular iron, sparing Fpn on the plasma membrane for export of excess iron. TfR, an iron uptake protein, is expected to decrease in an attempt to decrease iron uptake and maintain iron levels within normal limits because iron is an essential cofactor for several vital enzymes [39]. It is likely that iron is sequestered in ferritin and accumulates in autophagosomes and lysosomes. Our observations indicating co-localization of ferritin with LC3II-positive vesicles support this notion and suggest efficient delivery of ferritin by NCOA4 to phagosomes for fusion with lysosomes [40,41,42]. A small but significant increase in LC3II suggests an increase in autophagosomal activity, though not enough to limit its accumulation. Surprisingly, instead of accumulating in lysosomes, iron-loaded ferritin was released from cells in lysosomal membrane-enclosed vesicles.

The presence of excess ferritin in the RPE cell layer in-vivo and its dispersion to surrounding retinal layers presents several challenges. Ferritin-rich vesicles are secreted by the non-classical lysosomal secretory pathway [43] and probably gain entry into surrounding cells by ferritin receptors, such as scara-5 and TfR1 [44,45]. Internalization of ferritin is likely to increase the iron content of these cells and, in addition, other proteins and cellular debris enclosed in these vesicles. This pathway of dissemination of ferritin is of concern not only for AMD, but other ocular disorders associated with ferritin accumulation. The highly secretory nature of RPE cells facilitates this route, which is amplified by oxidative stress [46]. The release of iron-loaded ferritin probably protects these cells from iron-mediated damage but affects the fate of surrounding cells as lysosomal debris and other proteins form sub-RPE deposits typical of AMD [47]. The release of iron-loaded ferritin from RPE cells in lysosomal vesicles has not been reported so far in human AMD. If this is indeed the case and not an artifact of NaIO_3_ treatment, it would explain the critical role of iron in AMD and help in the development of therapeutic options aimed at preventing retinal iron accumulation.

Our observations suggest that the principal biochemical pathway affected by NaIO_3_ is decreased synthesis and maturation of cat-D, an aspartic protease responsible for the turnover of phagocytosed debris in RPE cells. Optimal activity of cat-D requires a low pH, and NaIO_3_ is known to increase lysosomal pH [48], reducing the activity of several lysosomal enzymes in addition to cat-D. A small but significant reduction in the synthesis of cat-D is also observed, which cannot be explained by reduced pH. It is likely that NaIO_3_ suppresses the transcription or increases the degradation of its mRNA, resulting in a significant reduction in its mRNA in NaIO_3_-treated cells. Consistent with these observations, a homozygous knockout mouse model of cat-D develops drusen deposits and retinal dysfunction by 12 months of age, and mice with deletions in cat-D that decrease the level of mature enzyme develop rapid and progressive loss of multiple retinal cell types, underscoring the significance of this enzyme in AMD pathogenesis [49]. However, neither mouse model developed choroidal neovascularization, a feature of wet AMD, indicating that impaired function of cat-D leads to dry AMD. Since cat-D activity is decreased by iron [17], it is likely that accumulation of iron-loaded ferritin and cat-D form a cycle, which, once initiated, feeds itself without additional insults.

In conclusion, this study indicates impaired turnover of iron-loaded ferritin due to impaired lysosomal function as the proximate cause of NaIO_3_-induced AMD in cell and animal models. Release of these vesicles in the extracellular milieu is likely to cause toxicity to the adjacent photoreceptors, resulting in retinal pathology mimicking AMD. Validation of these findings in human AMD will provide novel therapeutic options (Figure 7).

## Data Availability

Data is contained within the article and supplementary files.

## Supplementary Materials

The following are available online at https://www.mdpi.com/article/10.3390/antiox10081253/s1, Table S1. List of antibodies, Figure S1, Full immunoblots of images presented in the manuscript, Figure S2. Controls for the experiments in Figure 2E and Figure 3 in the manuscript, Figure S3. Controls for the experiment in Figure 5B (panels 5 & 6) in the manuscript.
