# Supplementary material for: Release of Iron-Loaded Ferritin in Sodium Iodate-Induced Model of Age Related Macular Degeneration: An In-Vitro and In-Vivo Study
Source: Antioxidants (Basel). 2021 Aug 5;10(8):1253. doi: 10.3390/antiox10081253 (PMC8389213; doi:10.3390/antiox10081253)
Supplement: Supplementary file 1 [file antioxidants-10-01253-s001.zip › antioxidants-1269832-supplementary.pdf]

Supplementary Files

Table S1. List of antibodies.

Primary Antibody	Host	Catalogue Number	Company	Dilution
Ferritin	Rb	F5012	Sigma-Aldrich, USA	WB-1:500<br>IHC:1:100
Ferritin	g	NBP1-06985	Novus Biologicals, USA	ICC-1:100
Transferrin Receptor	m	13-6800	Invitrogen, USA	WB-1:1000
pro-hepcidin	Rb	NBP1-59337	Novus Biologicals, USA	WB-1:500
Ferroportin	Rb	NBP1-21502	Novus Biologicals, USA	WB-1:500
LC3II	Rb	2775	Cell Signaling Technology, USA	WB-1:500<br>ICC-1:100
LAMP-1	Rb	ab24170	Abcam, USA	ICC-1:100
NCOA4	m	ab56356	Abcam, USA	WB-1:500
Cathepsin-D	m	sc-377299	SantaCruz, USA	WB-1:500
β-actin	m	MAB1501	Millipore Sigma, USA	WB-1:5000
**Secondary Antibody**				
HRP-conjugated secondary anti-mouse	Sh	NA931V	GE Healthcare, USA	WB-1:10000
HRP-conjugated secondary anti-rabbit	Dn	NA934V	GE Healthcare, USA	WB-1:10000
Anti-Rabbit IgG (H+L) cross adsorbed secondary antibody, Alexa Fluor 546	g	A11071	Invitrogen, USA	ICC-1:1000<br>IHC-1:1000
Anti-goat IgG (H+L) cross adsorbed secondary antibody, Alexa Fluor 488	Rb	A27012	Invitrogen, USA	ICC-1:1000

Rb: rabbit, m: mouse, Sh: sheep, Dn: donkey, g: goat; WB: Western blotting, ICC: immunocytochemistry, IHC: immunohistochemistry.

Figure S1. Full immunoblots of images presented in the manuscript. All gels are 12.5%.

Figure 1A

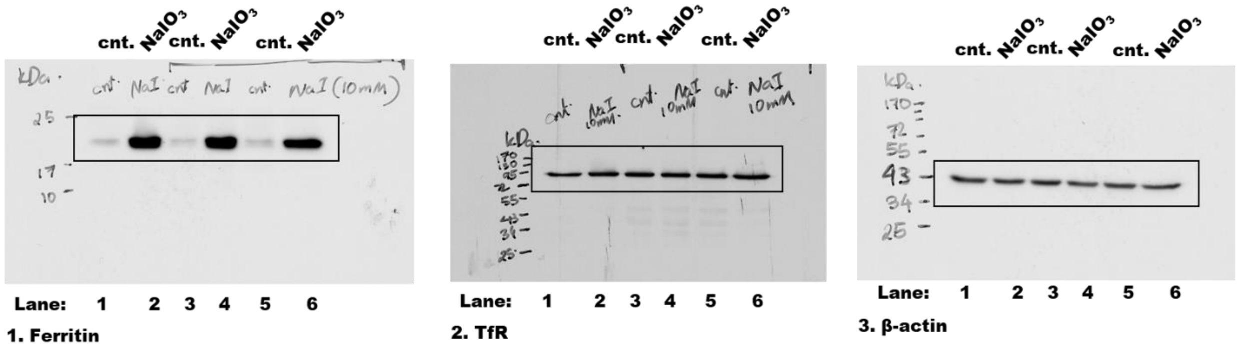

Membrane was cut at 25 kDa and the lower membrane (10–25 kDa) was probed for ferritin. The upper membrane (25–170 kDa) was probed for TfR and re-probed for β-actin.

Figure 1C

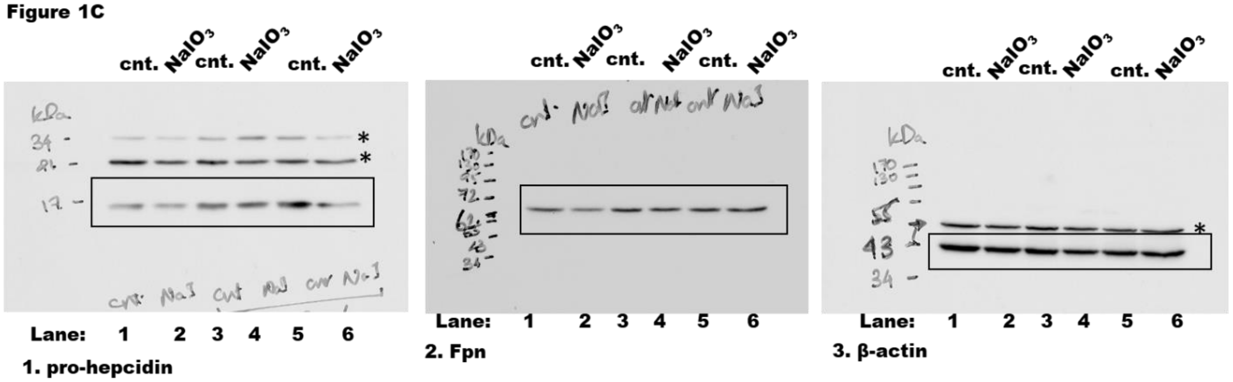

Membrane was cut at 34 kDa and the lower membrane (10–34 kDa) was probed for pro-hepcidin. The upper membrane (34–170 kDa) was probed for Fpn and re-probed for β-actin.* marks non-specific bands.

Figure 2A

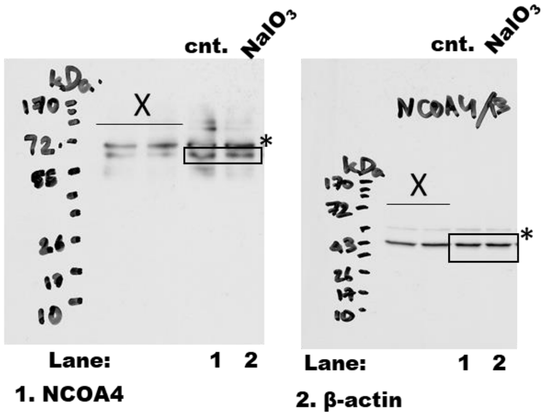

Membrane was probed for NCOA4 and re-probed for β-actin. X: irrelevant samples. * marks non-specific bands.

Figure 2C

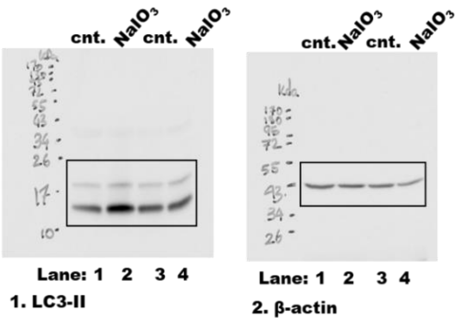

Membrane was probed for LC3II and re-probed for β-actin.

Figure 4B

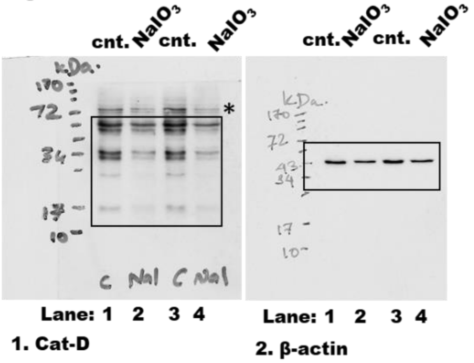

Membrane was probed for cat-D and re-probed for β-actin. * marks non-specific bands.

Figure 4E

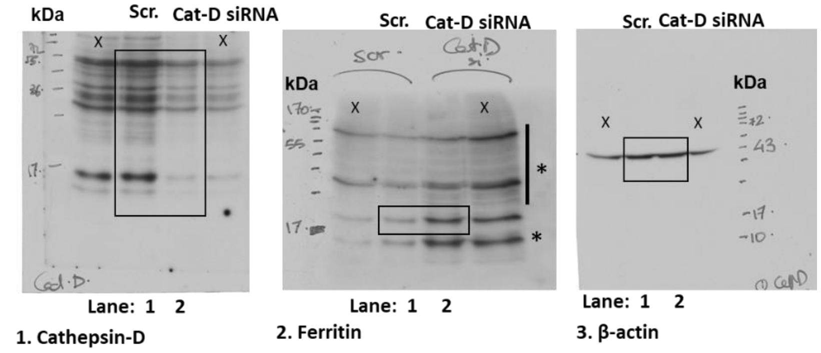

Membrane was probed for cat-D and re-probed for ferritin and β-actin. X: irrelevant samples. *non-specific bands.

Figure 5A

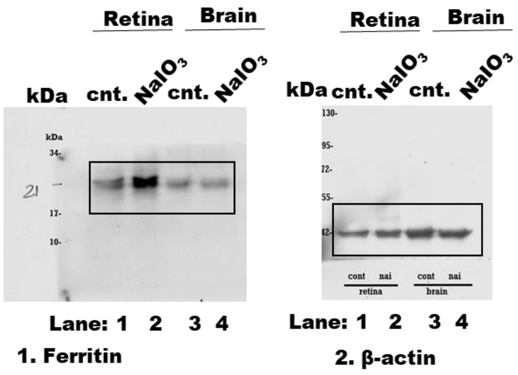

Membrane was cut at 34 kDa and the lower membrane (10–34 kDa) was probed for ferritin and the upper membrane (34–130 kDa) for β-actin. Non-specific binding was not detected.

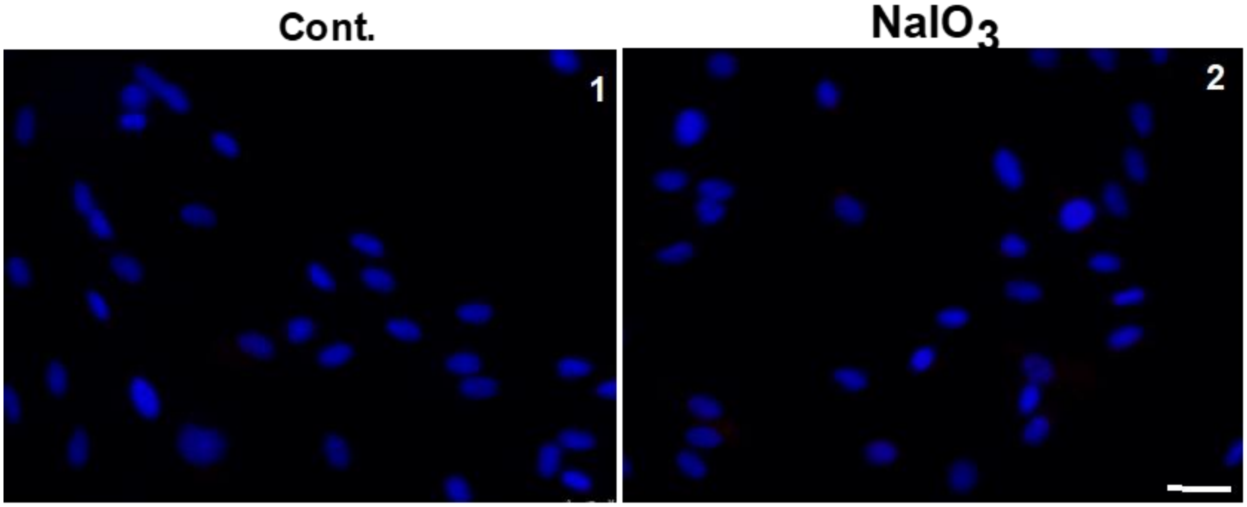

Figure S2. Controls for the experiments in Figure 2E and Figure 3 in the manuscript: ARPE-19 cells incubated with and without $NaIO_3$ (24 h) were reacted with both Anti-Rabbit IgG secondary antibody conjugated with Alexa Fluor 546 (red) and Anti-goat IgG secondary antibody conjugated with Alexa Fluor 488 (green). No reactivity was observed in either sample (panels 1 & 2). Images were acquired keeping all experimental parameters similar to Figure 2E and Figure 3. Scale bar: 25μm.

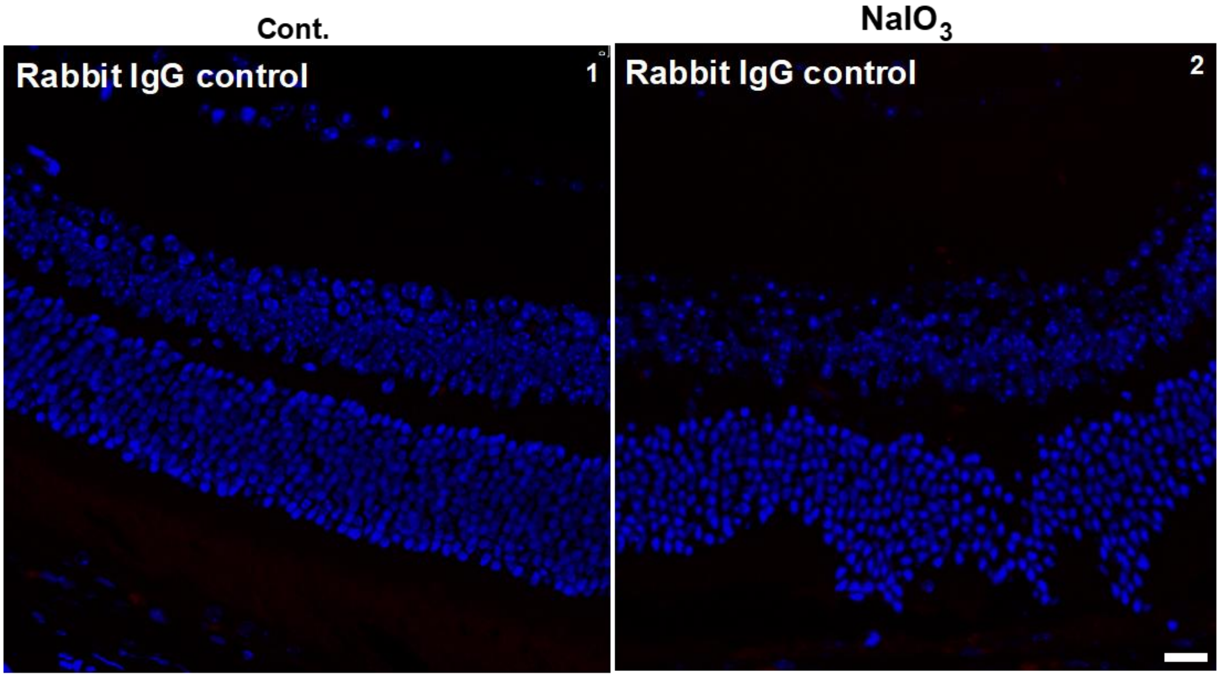

Figure S3. Controls for the experiment in Figure 5B (panels 5 & 6) in the manuscript. Serial sections of retinal tissue sections from Figure 5B were reacted with rabbit IgG and Anti-Rabbit IgG secondary antibody-Alexa Fluor 546 (red). The samples were processed and imaged simultaneously with the images shown in Figure 5B. No reactivity was observed in either sample (panels 1 & 2). Scale: 25μm.
